# Cancer-associated fibroblasts: multidimensional players in liver cancer

**DOI:** 10.3389/fonc.2025.1454546

**Published:** 2025-04-03

**Authors:** Yanyun Pan, Yuangang Qiu, Xinbin Zhou, Wei Mao, Xiaoming Xu

**Affiliations:** ^1^ Department of Cardiology, The First Affiliated Hospital of Zhejiang Chinese Medical University (Zhejiang Provincial Hospital of Chinese Medicine), Hangzhou, China; ^2^ Department of Cardiology, Affiliated Zhejiang Hospital, Zhejiang University School of Medicine, Zhejiang Key Laboratory of Integrative Chinese and Western Medicine for Diagnosis and Treatment of Circulatory Diseases, Hangzhou, China

**Keywords:** cancer-associated fibroblast, iCAF, myCAF, hepatocellular carcinoma, intrahepatic cholangiocarcinoma, tumor microenvironment

## Abstract

Cancer-associated fibroblasts (CAFs), the most abundant stromal cells in the tumor microenvironment (TME), control tumor growth through production and organization of the extracellular matrix (ECM) for a long time. However, the results from different studies that have focused on targeting CAFs to disturb tumor progression are extremely controversial. Recent studies using advanced single-cell RNA sequencing technology (scRNAseq) combined with multiple genetically engineered mouse models have identified diverse CAF subpopulations in the premalignant liver microenvironment (PME) of hepatocellular carcinoma (HCC) and TME of intrahepatic cholangiocarcinoma (ICC), providing a deeper understanding of the exact roles of each CAF subpopulation in cancer development. This review focuses on the specific protein markers, signaling pathways, and functions of various emerging CAF subclusters that contribute to the development of ICC and HCC. Elucidating the role and regulation of CAF subpopulations under different pathophysiological conditions will facilitate the discovery of new therapeutics that modulate CAF activity.

## Introduction

Cancer treatments have shifted from a tumor-centric view to a microenvironment-centric view because of the vital role of the tumor microenvironment (TME) or premalignant microenvironment (PME) in regulating tumor progression (1–4). The TME or PME comprises all non-cancerous host cells, including fibroblasts, endothelial cells, neurons, adipocytes, and immune cells, as well as its non-cellular components, including the extracellular matrix (ECM) and soluble products, such as chemokines, cytokines, growth factors, and extracellular vesicles (5, 6). The dynamic architecture of the TME or PME has a profound effect on tumor growth, which makes the interaction between malignant cells and their microenvironment a promising target for anti-cancer therapies, particularly those associated with excessive fibrosis.

Primary liver cancer remains among the deadliest human neoplasms worldwide owing to its poor prognosis and lack of effective therapeutics, despite numerous improvements in drugs and other medical therapies during the past decades (7). Hepatocellular carcinoma (HCC) and intrahepatic cholangiocarcinoma (ICC), which occur in the setting of chronic liver injury and inflammation, are the two main types of primary liver cancers, accounting for approximately 80% and 15% of cases, respectively (8). While most HCC at their early stage are characterized by fibrosis or cirrhosis formed by hepatic stellate cells (HSC) or cancer-associated fibroblasts (CAFs) in the PME, ICC is characterized by its desmoplastic TME in which CAFs are the main stromal components (9). CAFs are involved in tumor cell proliferation and metastasis, angiogenesis, immunomodulation, and chemoresistance via ECM deposition, interacting with other cells by secreting multiple cytokines and growth factors, or directly interacting with other cell types via ligand–receptor interactions in liver cancer (10), suggesting a key role of CAFs in tumor progression. Targeting CAFs may provide strong therapeutic effects for cancer treatment and biomarkers for diagnosis and prognosis. However, results obtained from distinct studies focused on disturbing ECM production or targeting CAF using genetically engineered mouse cancer models to treat cancer are contradictory, implying that the roles of CAFs in tumor progression are diverse and complex (11–14). For example, while CAF-produced type I collagen (COL-I) promotes the malignant phenotype of tumor cells, depletion of CAF leads to escape from immune surveillance and accelerates tumor progression in pancreatic cancer mouse models (15, 16). Recent breakthroughs in integrating cutting-edge methods involving high-resolution single-cell profiling coupled with sophisticated animal model manipulation strategies have shed light on various functional subsets belonging to the CAF population that contribute unique regulatory features towards shaping malignant cells or their microenvironment, offering new opportunities for targeting the trajectory of CAFs (17–21).

## Definition and origin of CAF

**Figure 1:**
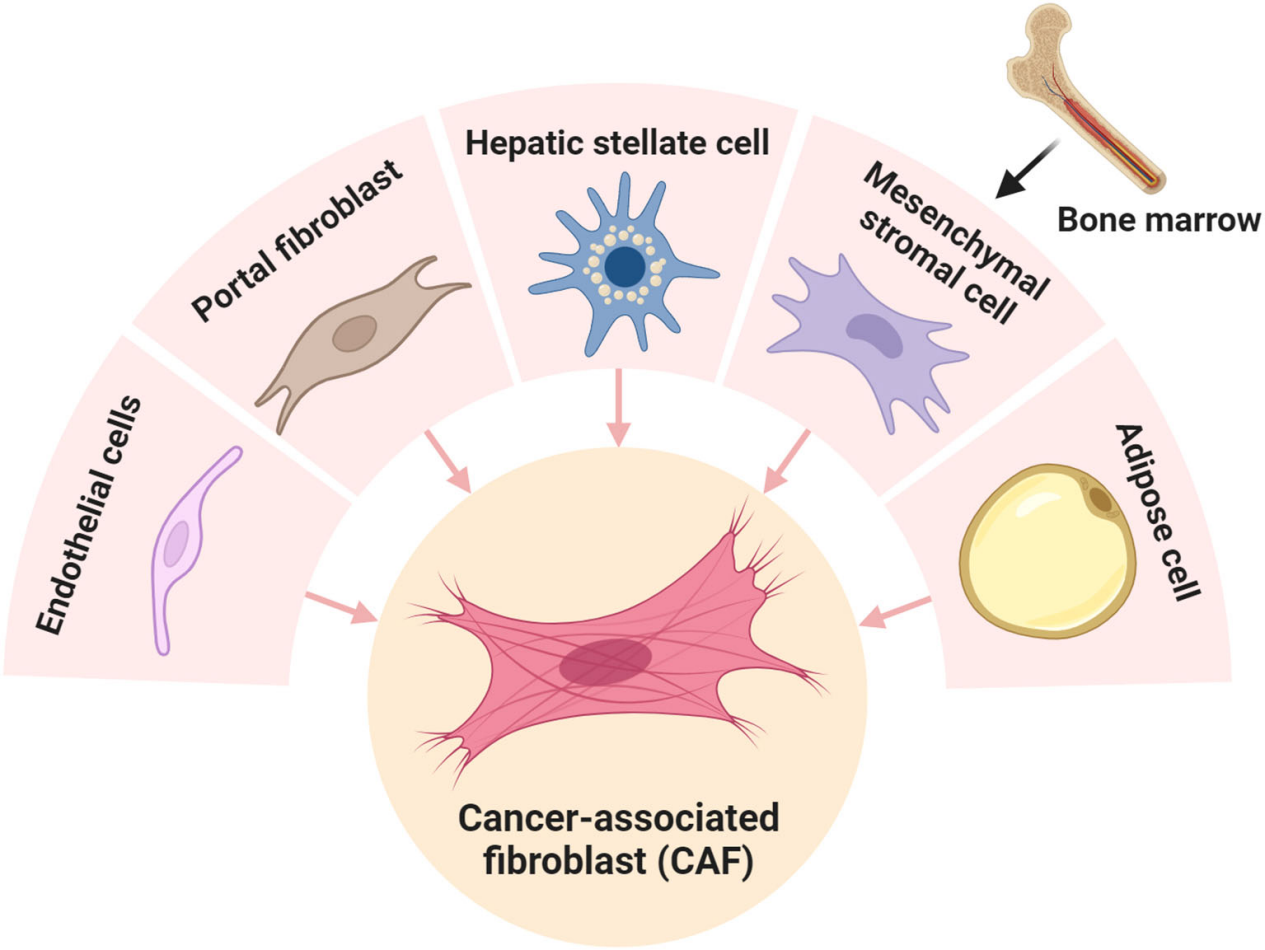
Cancer-associated fibroblasts (CAFs) originate from different cell types. CAFs can be driven from hepatic stellate cells (17, 19, 20, 27), portal fibroblast (21), endothelial cells (30), and bone marrow-derived mesenchymal stromal cells (31).

CAFs are thought to be a group of cells negative for epithelial, endothelial, and leukocyte markers, with an elongated spindle morphology and lack of mutations found within cancer cells (10). CAFs are activated fibroblasts characterized by higher expression of α-smooth muscle actin (αSMA) than quiescent fibroblasts or normal fibroblasts in the TME or PME (22, 23). Heterogeneity and plasticity, well-known hallmarks of CAFs, make only αSMA (+) available to define CAFs (24). However, the specific markers that can define all types of CAFs remain unknown because of the diverse cellular origins and functions of distinct CAF subclusters (24, 25). Most CAFs in the liver originate from HSCs which are quiescent fibroblasts constituting the native extracellular matrix (ECM) and maintaining liver homeostasis under basal circumstances (10, 24, 26). HSC can be activated and transformed into various subpopulations of CAFs in ICC and HCC after receiving stimuli from cancer cells or TME including cytokines and growth factors (17, 19, 20, 27). Another source of CAF in the liver are resident portal fibroblasts (PFs) with mesothelial characteristics that reside in portal spaces where they produce connective tissue containing the bile duct, portal vein, and artery (28). PF is transformed into a small subpopulation of CAFs, designated as mesCAFs with unknown functions in ICC (21). Interestingly, PF, rather than HSC, is the dominant origin of CAFs in colorectal cancer liver metastases, suggesting the complex origin and functions of CAFs in different TMEs (29). Apart from HSC and PF, other cells in the TME or PME can be alternative origins of CAF in the liver (25, 28, 29) (**Figure 1**). For example, a subpopulation of CAF is derived from endothelial cells, defined as CAF_EndMT_, which exhibits dual expression of canonical lineage markers of fibroblasts and endothelial cells, highlighting the possibility of its involvement in endothelial–mesenchymal transition (EMT) (30). In addition, CAF can also originate from adipose cells with unclear function (30) or be transformed from bone marrow (BM)-derived mesenchymal stromal cells (MSCs) that are recruited to primary breast tumors and lung metastases (31). However, whether BM-derived CAF exist in primary liver cancers remains unknown. Notably, although many new subpopulations of CAFs with specific markers have been identified using advanced scRNA-seq, the function of some CAFs remains unexplained. One possible reason is that these CAFs are in the intermediate state transited from the original CAFs and need to be differentiated into terminal effective fibroblasts to execute functions (18).

Lineage-tracking techniques combined with trajectory analyses and genetic ablation mouse models contribute to explaining how distinct subpopulations of CAFs are generated from their ancestors or transformed into each other, offering the possibility of targeting CAF transitions to anti-cancer (10, 17, 18, 20, 32–34). Recently, CAF transitions have been found to play an important role in liver fibrosis and HCC development. Through scRNA-seq in conjunction with pathway analysis and genetic ablation, two subpopulations of fibroblasts were identified in PME during HCC progression, termed myofibroblastic CAF (myCAF) and cytokine- and growth factor-expressing CAF (cyCAF) (20). The former was enriched in ECM-related molecules and pathways, whereas cytokines and growth factors were highly expressed in the latter. CyCAF is decreased and myCAF is increased in mice with liver fibrosis and cirrhosis, which promotes HCC development, whereas the shift in cyCAF-myCAF balance towards cyCAF inhibits HCC, suggesting that cyCAF–myCAF transitions play a fundamental role in orchestrating hepatocarcinogenesis. By investigating cellular heterogeneity and transcriptomic profiles during ICC initiation and progression (19), another study showed that while cytokine-producing inflammatory CAF (iCAF) are enriched in the early stage of ICC, myCAFs accumulate in the late stage of ICC, implying that the iCAF–myCAF transition promotes ICC progression. These findings suggest that CAF subpopulations and their functions vary among different malignant states, cancer types, and genetic contexts (17–20).

## Distinct subpopulations of CAFs in primary liver cancer

Different subpopulations of CAFs perform either tumor-supporting or -restraining roles by interacting with tumor cells, immune cells, and other components within the TME and producing multiple ECM proteins, cytokines, chemokines, growth factors, and exosomes (2, 17, 35–39). Given the sophisticated role of CAFs in regulating cancer progression, it is necessary to understand the features of various CAF clusters in the TME.

### CAF subpopulations of ICC

MyCAF and iCAF are two canonical CAF subpopulations in ICC: the former produces ECM and the latter secretes cytokines and growth factors (40–42). With improvements in scRNA-seq and a deeper understanding of CAF characteristics, CAFs can be classified into six subpopulations in ICC (**Table 1**): myCAF, iCAF, vascular CAF (vCAF), antigen-presenting CAF (apCAF), lipid metabolism and processing CAF (lpCAF), and epithelial CAF (eCAF) (27). All six subsets exhibited robust expression of canonical fibroblast markers like *ACTA2* (α-SMA), *COL1A2*, and *PDGFRβ*, confirming their identity as fibroblasts; nevertheless, distinctive transcriptomic profiles were discernible among these groups. These subpopulations primarily derive from HSC, the quiescent or normal fibroblast in the liver parenchyma upon stimulation via various signals originating from neighboring cells (tumor cells or tumor-associated macrophage (TAM)) (17, 19, 39).

**Table 1:** CAF subpopulations of intrahepatic cholangiocarcinoma (ICC) and hepatocellular carcinoma (HCC).

Cancer types	Subtype of CAFs	Enriched pathways	Function	Highly expressed marker genes
**ICC**	myCAF	ECM production and fibril organization (19, 43, 49)	Promoting ICC progression at the late tumor stage, EMT, and immunosuppression (19, 43, 49)	COL1A1, SERPINF1 (24, 26, 27, 41, 42)
	iCAF	ECM, inflammatory response regulation, and complement activation (21, 27)	Immune modulation, recruitment of lymphocytes, promoting ICC progression at the early tumor stage (21, 27)	FBLN1, IGFI, CXCL1, IGFBP6, SLPI, SAA1 (21, 27)
	vCAF	Muscle contraction, responses to hypoxia, and mesenchymal cell proliferation (27)	Promote tumor cell stemness and growth (27)	CD146 (MCAM), MYH11, GJA4, and RGS5 (27)
	apCAF	Leukocyte cell-cell adhesion, response to IFN-g, antigen processing, and antigen presentation via MHC-II (27)	Regulating tumor immunity (27)	CD74, HLA-DRA, and HLA-DRB1 (27)
	lpCAF	Lipid metabolism and processing (18, 27)	Potential role in EMT (18)	APOA2, FABP1, FABP4, and FRZB (27)
	eCAF	Amoeba-like cell movement and epithelial cell migration related to angiogenesis (27)	Under investigation whether it is an independent subcluster	KRT19, KRT8, and SAA1 (27)
**HCC**	myCAFs	ECM-related molecules and pathways and EMT (17, 20)	Promoting HCC progression via COL-I-DDR1 pathway (20, 46)	COL1A1, COL1A2, COL5A1, COL6A3, POSTN, DCN, FAP, SPP1, TIMP1 (17, 20)
	cyCAF	Cytokines and growth factors (20)	Inhibiting HCC progression via HGF-MET axis (20)	RGS5, COLEC11, ECM1, HGF (20)
	vCAF	Vascular smooth muscle contraction and response to calcium ions (17)	Neoangiogenesis (17)	MYH11, MUSTN1, and MCAM (17)
	apCAFs	MHC-class-II protein complex and antigen processing and presentation (17)	Potential role in macrophage polarization and T-cell recruitment (17)	CD74, HLA-DRA, and CCL5 (17)
	lpCAFs	Protein-lipid complex remodeling and hallmark of fatty acid metabolism (17)	Recruitment of CD33+ myeloid-derived suppressor cells (17)	APOA1 and APOC1 (17)
	lpmCAFs	ECM, cholesterol metabolism, fatty acid metabolism and reactive oxygen species (17, 20)	Recruitment of CD33+ myeloid-derived suppressor cells and promoting HCC progression (17)	COL6A3 and COL1A1,CD36 and STEAP4 (17)

MyCAF is a typical example of CAFs found in tumors, characterized by the involvement of specific genes linked to ECM production and fibril organization, such as *COl1A1* and *SERPINF1* (24, 26, 27, 43, 44). As a result, it receives an additional label for the matrix CAF (mCAF) (27, 45). MyCAFs promote ICC proliferation and are associated with intraneural invasion. High expression of myCAF signature genes was correlated with poor survival and displayed a trend toward higher recurrence in the Sia cohort (46). Type I collagen (COL-I) is enriched in myCAF while its cognate receptor DDR1, is expressed in tumor cells, suggesting a link between myCAF and tumor cells via COL-I-DDR1 interaction, which has been verified in PDAC where COL-I-DDR1 axis orchestrates tumor growth and metastasis via regulating macropinocytosis, a nutrient scavenging pathway, and mitochondria metabolism (1, 2, 21, 47, 48). However, despite the ablation of COL-I in either fibroblasts or all liver cells in mouse ICC models using hydrodynamic delivery of oncogenic drivers into hepatocytes reducing stiffness and mechanosensitive signals, loss of COL-I does not prevent tumor growth, implying that COL1-DDR1 signaling is not essential for ICC development (21). Deletion of the other most upregulated gene in myCAF, hyaluronan synthase 2 (Has2), significantly reduces ICC tumor growth with an unknown mechanism due to the multiple hyaluronan receptors and multiple cell types of Has2-expressing myCAF (21). However, it is difficult to rule out DDR1’s involvement in ICC development because hydrodynamic transfection cannot be applied to study tumors originating from hepatic stem cells or biliary epithelial cells and cannot create a fibrotic microenvironment, a typical feature of human ICC (49). Another possible explanation of why myCAF-produced COL-I might not contribute significantly to ICC growth involves the apparent abundance of myCAF observed recently in the late stage of ICC combined with driving ICC progression via Igf1/Igfr signaling (19). In addition, myCAF is found to potentially promote epithelial mesenchymal transition (EMT) and immunosuppression via TGFβ signaling (45, 50, 51). Together, these results suggest that myCAF is an oncogenic biomarker for advanced ICC stages and poor prognosis, and targeting Has2, TGFβ, or other key regulators that can disturb the interaction between myCAF and other cell types may offer strong therapeutic effects. PEGPH20, a PEGylated human hyaluronidase that can degrade excessive stromal hyaluronan, remodels the TME and consistently achieves objective tumor responses in PDAC (52). Galunisertib, a small-molecule inhibitor of TGFβ receptor I kinase that blocks TGFβ signaling, has been under Phase I/II trials for multiple cancer types (26, 53). Whether PEGPH20 and Galunisertib could inhibit myCAF in ICC should be further investigated.

iCAF, another canonical subpopulation of CAFs in the TME of ICC, express high levels of *FBLN1*, *IGFI*, *CXCL1*, *IGFBP6*, *SLPI*, *SAA1*, and complement genes (*C3* and *C7*). Gene ontology (GO) terms enriched for this subcluster are related to ECM, inflammatory response regulation, and complement activation, indicating that iCAF may engage in immune modulation (27). Based on bulk RNA sequencing data from TCGA gastric cohort, the expression levels of gene markers for iCAFs are associated with the abundance of lymphocytes, suggesting that iCAFs play an important role in the recruitment of lymphocytes (54). The main subpopulation of CAFs residing in lymphoid nodule-like structures are iCAFs, which are located around CD8^+^ and PD1^+^ T cells, indicating that iCAFs are involved in regulating T cells (54). Unlike myCAF, iCAF is enriched in early stage ICC and promotes ICC development through the hepatocyte growth factor (HGF)-MET (HGF receptor) axis as a key tumor-promoting ligand–receptor pair, directly linking iCAF to tumor cells via ERK-mediated tumor cell proliferation (21). This has been validated in HGF-ablated and MET-ablated mouse ICC models, as well as in CellPhoneDB, which is a publicly available repository of curated receptors, ligands, and their interactions (21, 55). However, the mechanism by which iCAF interacts with lymphocytes in ICC required further investigation. These results suggest that iCAF is an oncogenic biomarker of the early ICC stage and is correlated with a suppressed immune environment. Xentuzumab, a humanized monoclonal antibody targeting IGF1 and IGF2, has been in Phase I and II trials for multiple cancers. It showed preliminary antitumor activity in a heavily pretreated population (56). However, whether the therapeutic effects of Xentuzumab are derived from blocking iCAF remains unknown.

apCAFs express major histocompatibility complex II (MHC-II) genes such as CD74, HLA-DRA, and HLA-DRB1. The GO terms enriched in this subcluster are related to leukocyte cell–cell adhesion, response to IFN-γ, antigen processing, and antigen presentation via MHC-II, suggesting a function in regulating tumor immunity (27). apCAFs were first identified in pancreatic ductal adenocarcinoma (PDAC), which can present antigens to CD4^+^ T cells (57). By integrating multiple scRNA-seq studies and performing robust lineage-tracing assays, apCAFs were found to be derived from mesothelial cells during pancreatic cancer progression (58). This mesothelial cell-to-apCAF transition is induced by interleukin-1 and TGFβ by downregulating mesothelial features and upregulating fibroblastic features. However, a pan-cancer single-cell analysis revealed that apCAF, presenting TAM (such as *MAFB* and *SPI1*) and CAF (such as *MYLK*) regulons, is at an intermediate position during the TAM to myCAF transition. Further investigations are needed to determine whether apCAF is located in an intermediated state, where it originates, and how it contributes to tumor progression in ICC.

vCAF are the most abundant fibroblast population characterized by microvasculature signature genes such as *CD146 (MCAM)*, *MYH11*, *GJA4*, and *RGS5*, as well as inflammatory chemokines such as *IL-6* and *CCL8* (27). vCAFs are significantly enriched in pathways related to muscle contraction, responses to hypoxia, and mesenchymal cell proliferation, all of which align with their microvasculature signatures. Of note, vCAFs are located in the tumor core and microvascular area and promote tumor cell stemness and growth through secretion of IL-6 which induces significant epigenetic alterations in tumor cells, particularly upregulating enhancer of zeste homolog 2 (EZH2) (27), suggesting that there is an intense interaction between vCAF and ICC tumor cells, and the anti-IL6 pathway is a potential treatment for ICC. However, IL-6 is also important for the differentiation of naïve CD4^+^ T cells into Th17 cells and for protecting T cells from apoptosis (59). Therefore, improving the therapeutic effects by targeting vCAF requires further investigation.

lpCAFs are mainly derived from adjacent ICC tumor tissues and express high levels of lipid metabolism- and processing-related genes, including *APOA2*, *FABP1*, *FABP4*, and *FRZB* (27). lpCAFs are found to express marker genes similar to adipogenic CAFs, which are abundant in genes associated with EMT across various cancers analyzed via single-cell sequencing analysis (18). Whether lpCAFs also contribute to EMT in ICC requires further investigation. eCAFs express both epithelium-specific marker genes, such as *KRT19*, *KRT8*, and *SAA1*, and activated fibroblast makers (27). The gene expression profile of eCAFs includes activities associated with amoeba-like cell movement and epithelial cell migration signals related to angiogenesis. However, it is still under investigation whether eCAFs should be considered an independent subgroup because they represent epithelial/CAF doublets (45).

## Subpopulations of fibroblasts in PME and TME during HCC progression

Most HCCs arise in the context of fibrosis or cirrhosis, including those associated with non-alcoholic steatohepatitis (NASH), despite the absence of overt cirrhosis in 30%–50% of NASH-associated HCC (60). Emerging studies combining genetic CAF activation, inhibition, or depletion approaches in various mouse models of spontaneous, carcinogen-induced, oncogene-induced, and NASH-induced HCC have demonstrated that CAFs exert an overall HCC-promoting role (20). Co-implantation of CAFs with HCC cells also enhances HCC progression (17). However, deletion of CAF in mice with established tumors does not affect HCC development, suggesting that fibroblasts predominantly promote tumor growth by modulating PME and the early stages of hepatocarcinogenesis. A global view of fibroblasts in PME and TME during HCC progression is necessary and meaningful to deeply understand their precise role.

Integration of scRNA-seq of fibroblasts from various mouse models with fibrotic liver, normal and cirrhotic human livers, and pathway analyses, fibroblasts in PME can also be divided into two typical subpopulations, myCAF and cyCAF (20) (**Table 1**). Similar to myCAF in ICC, myCAF is characterized by enrichment of ECM-related molecules and pathways, whereas cyCAF, close to iCAF of ICC, is a cluster of quiescent to weakly activated CAFs enriched in genes and pathways related to cytokines and growth factors. These two clusters transition to each other to regulate hepatocarcinogenesis (20). The shift in cyCAF/myCAF balance towards myCAFs exhibits increased HCC numbers and towards cyCAFs exhibits decreased HCC numbers, implying that the ratio of cyCAF/myCAF is a strong prognostic indicator, which requires a wide examination of human HCC tumor specimens and survival for verification. Trajectory and pseudotime analyses of liver fibrosis models revealed that myCAFs might be derived from cyCAFs. However, given that activated CAFs may revert to quiescence, it is possible that myCAFs can transition to cyCAFs or shuttle back and forth, especially when tumor progression is dynamic. In addition, both clusters can strongly interact with hepatocytes and affect biology and tumorigenesis. MyCAF-secreted COL-I promotes proliferation and tumor development through increased stiffness and TAZ activation in pretumoral hepatocytes and through the activation of DDR1 in established tumors. Notably, consistent with the results obtained from PDAC (48), matrix metalloproteinase-cleaved COL-I rather than intact COL-I promoted DDR1 activity in HCC tumor cells, suggesting that targeting the components of cleaved COL-I-DDR1 signaling may provide an alternative therapeutic strategy for anti-HCC (20, 48). Indeed, DDR1 inhibitors have been widely used in preclinical tumor models. For example, 7rh a selective inhibitor of DDR1 has been shown strong inhibitory effects in tumor cell proliferation of breast cancer, pancreatic cancer, and gastric cancer (48, 61, 62). In contrast, cyCAF-produced HGF activates MET primarily expressed in hepatocytes to suppress hepatocarcinogenesis (20). However, future studies should focus on the mechanisms by which DDR1 activation accelerates and the HGF–MET axis decelerates HCC tumor growth.

Unlike ICC, in which CAF subpopulations from different studies are relatively consistent, CAF subpopulations vary in HCC studies by distinct research groups (17, 20). In addition to the classification mentioned above, the CAFs subpopulation in the TME of HCC can be grouped into five common subtypes: vCAF, myCAFs, lipid processing (lp)-myCAFs (lpmCAFs), lpCAFs, and apCAFs by scRNA-seq of human and mouse HCC tumors (**Table 1**). iCAF is not included. All five subpopulations expressed canonical fibroblast markers such as *ACTA2*, *COL1A2*, and *COL1A1*. Notably, apart from lpmCAFs, the characteristics of the other four CAF subclusters were similar to their ICC counterparts (**Table 1**). lpmCAFs expressed both ECM-related genes (such as *COL6A3* and *COL1A1*) and lipid metabolism-related genes (such as CD36 and STEAP4) (**Table 1**). The GO and KEGG terms enriched for this cluster were related to ECM, cholesterol metabolism, fatty acid metabolism, and reactive oxygen species pathways, implying that this subpopulation is involved in both ECM and cholesterol metabolism. CD36 mediates oxidized LDL uptake-dependent expression of macrophage migration inhibitory factor (MIF) via the lipid peroxidation/p38/CEBPs axis in lpmCAFs, which may recruit CD33^+^ myeloid-derived suppressor cells (MDSCs) in an MIF- and CD74-dependent manner to promote HCC. lpmCAFs may be located in the intermediate state and transit to vCAFs, lpCAFs, and apCAFs, as determined by trajectory analysis. Infiltrating lpCAFs, especially in tumor tissues, can recruit CD33^+^ myeloid-derived suppressor cells to induce immunosuppression and correlate with poor prognosis in patients with HCC. Taken together, current research into subpopulations of HCC CAFs remains at the level of cell classification, and very little is known about the exact functions of each subset during tumor progression.

In addition, CAFs are key producers of vascular endothelial growth factor (VEGF), which stimulates the formation of new blood vessels to support tumor growth. CAFs can also produce angiopoietins and matrix metalloproteinases (MMPs), which further enhance angiogenesis by promoting vessel sprouting and degrading physical barriers. Moreover, CAFs secrete platelet-derived growth factor (PDGF), fibroblast growth factor (FGF), and stromal-derived factor 1 (SDF-1/CXCL12), which recruit endothelial progenitor cells and promote vascularization.

## Future directions and conclusions

Although CAF have been considered a potential therapeutic target for a long time, the results obtained from targeting CAF to treat cancer in preclinical models are extremely controversial. Advanced scRNA-seq combined with various genetically engineered mouse models and clinical species identify distinct CAF subpopulations displaying either tumor-promoting or -suppressing roles, which offers possibilities to target specific subclusters to cure cancer (17–21, 27, 43, 45). The tumor-promoting functions of CAF subpopulations make them promising targets for anticancer therapy, providing attractive potential therapeutic strategies that can be categorized as blockage of CAF subpopulation recruitment and activation, depletion of CAF populations, and blockage of interactions between CAF subpopulations and tumor cells or immune cells. To accomplish this, more efforts should be made to elucidate the molecular mechanisms by which CAF subpopulations influence tumor progression. Trajectory analysis revealed the origin of distinct subpopulations of CAF and subpopulations that are located in the intermediate state. However, further investigation is needed to confirm the origin of each subpopulation and the exact function of the intermediate clusters using new genetically engineered mouse cancer models. The mechanism by which the CAF transitions occur is still not fully understood. However, the dynamics of DNA methylation, histone acetylation, and histone methylation in several types of cancer have been found to play a potential role in CAF activation and transitions (63–67). For example, the loss of H3K27me3 activates CAFs to promote stem cell niche formation and tumor growth (67). In contrast to cancer cells carrying numerous mutations, CAFs possess greater genetic stability, another hallmark of CAFs (68). Based on this, it is necessary to investigate the epigenetic regulation of CAF transitions and CAF origin in different contexts, which will help develop novel strategies to interpose the shuttle between distinct CAF clusters and understand the origin and diversity of CAFs, as well as bring practical therapeutic efficacy and slight side effects. Notably, even though several CAF subpopulations are similar between ICC and HCC, the specific markers are not the same, which means targeting the “same” CAF subpopulation in the same organ may use distinct inhibitors in different cancers. Whether there is a long way to go to develop specific regulations to change the composition of CAF subpopulations in PME or TME for anticancer treatment.

Although scRNA-seq greatly advances our understanding of tumor cell heterogeneity, it has some limitations. Spatial and morphological information is lost after tissue dissociation into a single-cell suspension, making it difficult to investigate the tumor spatial architecture. Recently developed spatial transcriptomics technology (STT) may overcome these limitations because it provides information on spatial TME characteristics from non-tumor to leading-edge tumor regions of primary liver cancers (69). Additionally, as compared to studies of a single omics type, multi-omics that are capable of interrogating entire pools of transcripts, proteins, and metabolites, as well as the genome, offer the opportunity to understand the flow of information, from the functional consequences or relevant interactions. However, the importance of the tremendous amount of data produced by STT or multi-omics in practice requires the development of new preclinical models for verification. Another major challenge in current CAF research is nomenclature. Unified naming rules to define CAF subpopulations or other cell types with specific markers and developmental trajectories will benefit all researchers in understanding the unique aspects of each cancer type, avoid repetitive research, and allow researchers to easily follow the findings from different study groups. A simple and efficient solution is to reach expert consensus. In addition, accurate recording of CAF subpopulations in clinical specimens limits the application of targeting CAF subpopulations in clinical settings. This depends on high-quality antibodies against marker proteins of each CAF subcluster, which in many cases is lacking. Technology for multiplexed mRNA probes characterized by high sensitivity and specificity is developing rapidly and may offer a better and more flexible solution than antibody-based methods. Moreover, identifying specific biomarkers for each CAF subpopulation and combined inhibition of the CAF subpopulation with immunotherapy or chemotherapy will expedite the application of CAFs in liver cancer treatment.
